# Association Between Prognostic Nutritional Index and Prognosis in Patients With Heart Failure: A Meta-Analysis

**DOI:** 10.3389/fcvm.2022.918566

**Published:** 2022-06-10

**Authors:** Mei-Yu Chen, Jiang-Xiong Wen, Mei-Ting Lu, Xiang-Yu Jian, Xiao-Liang Wan, Zhi-Wen Xu, Jian-Qiu Liang, Jian-Di Wu

**Affiliations:** ^1^Department of General Medicine, The Second People's Hospital of Foshan, Foshan, China; ^2^Department of Cardiology, The Second People's Hospital of Foshan, Foshan, China

**Keywords:** prognostic nutritional index, heart failure, all-cause mortality, cardiovascular disease, prognosis

## Abstract

**Background:**

The prognostic nutritional index (PNI) has been proposed as a marker of malnutrition and associated with the prognosis of cardiovascular disease. However, whether PNI can serve as a potential biomarker for the prognosis of heart failure (HF) upon those established risk factors were still controversial. This meta-analysis aimed to generate comprehensive evidence on the prognostic value of PNI in patients with HF.

**Methods:**

Multiple databases (PubMed, Embase, the Cochrane Library, and Google Scholar) were searched for related studies up to January 31, 2022. Observational studies accessed associations between PNI levels and the prognosis in patients with HF were included for meta-analysis. The hazard ratios (HRs) and 95% confidence intervals (CI) were calculated.

**Results:**

Fourteen studies, comprising 19,605 patients with HF were included for meta-analysis. The median follow-up duration was 18.5 months. Compared with those with higher PNI (normal nutritional status), patients with HF with lower PNI (malnourished) were associated with a higher risk of all-cause mortality (HR 1.53, 95% CI 1.27–1.85) and composite major adverse cardiac outcomes (MACEs; HR 2.26, 95% CI 1.54–3.31) in the multivariable-adjusted model. Furthermore, when PNI was defined as per 1 increment as a continuous metric, higher PNI was associated with a decrease in all-cause mortality (per 1 increment of PNI: HR 0.94, 95% CI 0.88–0.96) and MACEs (per 1 increment of PNI: HR 0.97, 95% CI 0.95–0.98).

**Conclusions:**

The PNI can serve as an easily calculated bedside “malnutrition-inflammation” biomarker in HF. Lower PNI was associated with a worse prognosis in patients with HF.

## Introduction

Heart failure (HF) is the end state of various heart diseases. It was estimated that over 37.7 million individuals were affected with HF worldwide (1, 2). During the past decades, there have been significant improvements in the management of HF, especially with the use of new drugs, including angiotensin receptor neprilysin inhibitors and sodium-glucose cotransporter-2 inhibitors, and the prognosis of HF had been improved (3). However, the risk of mortality and re-hospitalization are still high, which become a growing public health burden worldwide (4–6). Therefore, it was important to further address novel pathogenic mechanisms, prognostic factors, and treatment modalities in HF (7–9).

Malnutrition is an important prognostic factor in HF. It was estimated that up to 50% of patients with HF were malnourished (10). Current guidelines for the management of HF recommend assessment of nutritional status in chronic HF (3, 11); however, there is no consensus on the tools or metrics for measuring malnutrition. Recently, a novel and simple metric, the prognostic nutritional index (PNI), had been proposed to evaluate the nutritional status in multiple clinical settings, including cancers, and post-operative pneumonia (12, 13). Recent studies also showed that lower PNI is associated with an increased risk of mortality or major adverse cardiac events (MACEs) in patients with acute or chronic HF (14–18). However, whether PNI can serve as a potential biomarker for the prognosis of HF upon those established risk factors were still controversial.

Herein, we conducted a systematic review and meta-analysis of observational studies to generate comprehensive evidence on the prognostic value of PNI in patients with HF, and further explore whether such a relationship is modified by other risk factors.

## Materials and Methods

### Search Strategies and Study Selection Criteria

This study was performed under the guideline of the Meta-analysis of Observational Studies in Epidemiology (MOOSE) Group (19). We searched multiple databases (PubMed, Embase, the Cochrane Library, and Google Scholar) from inception until January 31, 2022. The search was developed by combining the MeSH heading and text strategies, with the terms “prognostic nutritional index,” “PNI” or “malnutrition” and “heart failure,” “cardiac failure,” “myocardial failure,” “cardiac dysfunction,” or “myocardial dysfunction.” The search was limited to human studies and writing in English or Chinese. We also manually checked the reference lists of the included studies to identify other potential related articles.

The inclusion criteria for meta-analysis were: (1) observational studies involving adult participants with a diagnosis of HF; (2) the PNI was recorded at enrolment; and (3) the association between PNI score and the prognosis [including all-cause mortality or composite major adverse cardiac outcomes (MACEs)] of HF were reported during follow-up. The PNI value was calculated as: PNI = 10 × serum albumin concentration (g/d6) + 0.005 × total lymphocyte count (per mm^3^) (20).

The exclusion criteria were: (1) cross-sectional studies without follow-up evaluation; (2) reported other nutritional indexes but not PNI; and (3) duplicated publications from the same observational studies in such situations, only the most recently published data were included for analysis.

### Data Extraction and Study Quality Evaluation

Two researchers (CM and JW) conducted the literature searching and item screening independently. Potentially related articles were reviewed and study information was extracted into a predefined form. We evaluated the quality of the included studies according to NOS (the Newcastle–Ottawa Quality Assessment Scale for cohort studies), which assesses the selection, comparability, and exposure/outcome, respectively (21). Up to a maximum of nine points can be awarded in NOS and we defined studies were graded in quality as poor (<4 points), fair (4–6 points), or good (≥7 points), respectively (7, 22).

### Statistical Analysis

We assessed whether PNI was associated with the prognosis in patients with HF. The primary outcome was all-cause mortality. The secondary outcome was MACEs, including all-cause mortality, and HF re-hospitalization or all-cause re-hospitalization. The association of the outcomes and PNI value was reported in different ways in the included studies, such as per 1 increment as a continuous metric; or as normal/malnutrition in classified trait. Therefore, we calculated the hazard ratios (HRs) for per 1 score increment in PNI level, and also pooled data as malnutritional vs. normal nutritional status, respectively. To explore the effect of confounders on the estimated risks, unadjusted and multivariable-adjusted relative risks were both calculated. If multiple results were reported from different statistical adjustment models, we extracted the data which had adjusted the maximal number of confounders for analysis.

The HRs and their corresponding standard errors (SEs) were pooled by the inverse variance approach. In case outcomes were presented as odds ratios (ORs) or risk ratios (RRs), they were used as an approximate HR in meta-analysis (7, 22). We use the *I*^2^ statistics to test heterogeneity, and an *I*^2^ value of >50% or *P* for heterogeneity <0.1 was considered as statistically significant heterogeneity. A random-effects model was used to combine the pooled estimates if significant heterogeneity was observed. Otherwise, a fix-effects model was used.

Due to the limited number of available studies, we did not perform subgroup analyses for the association between PNI and the prognosis in patients with HF. The sensitivity analyses were conducted by interchanging the statistical models (random-effects models vs. fixed-effects models) or by omitting one study at a time. Publication bias was evaluated by inspecting the funnel plot for the outcomes. Due to the limited number of studies for each analysis (all *n* < 10), we did not further perform Begg's test or Egger's test to explore the publication bias. All the meta-analyses were conducted using RevMan 5.3 (The Cochrane Collaboration, Copenhagen, Denmark). We consider a *P*-value < 0.05 as statistically significant.

## Results

### Studies Retrieved and Characteristics

**Figure 1:**
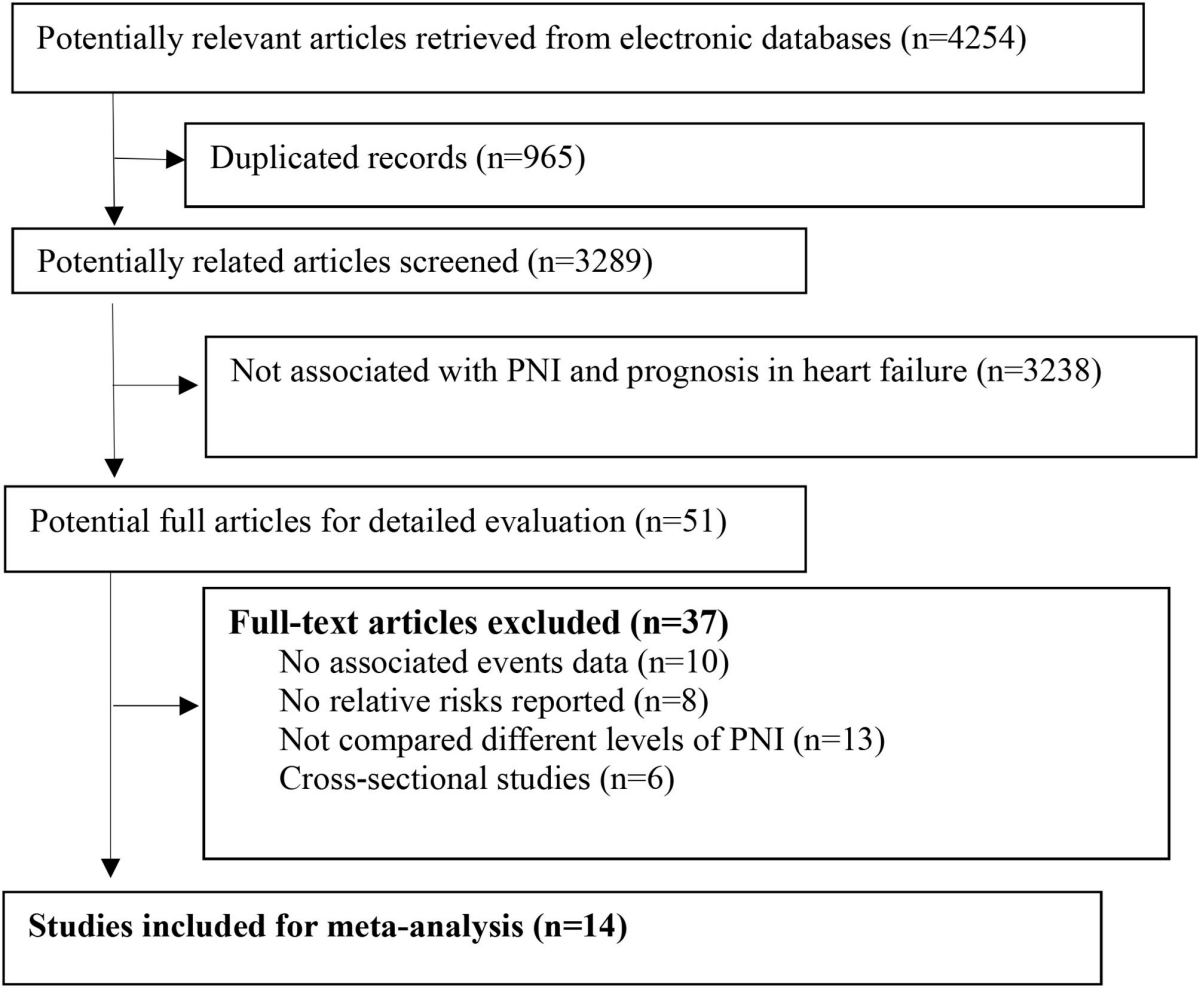
Flow of papers through review. PNI, prognostic nutritional index.

We retrieved 4,254 related articles from the databases. After deleting the duplicate items, two authors independently screened the titles and abstracts and reviewed the full text of 51 articles (Figure 1). Finally, according to the predefined inclusion and exclusion criteria, we included 14 studies comprising 19,605 patients with HF for analysis (14–18, 20, 23–30). The median follow-up duration was 18.5 months. The main baseline characteristics of the included studies are presented in Table 1. Most of the included studies adjusted the important confounders for the prognosis of HF (Supplementary File 1). According to the predefined NOS assessment, we graded 4 studies with fair study quality and 10 studies with good quality (Supplementary File 2).

**Table 1:** Characteristics of studies evaluated the association between prognostic nutritional index (PNI) and the outcomes in patients with heart failure (HF).

**Study**	**Country/region**	**Study design**	**Cohort characteristics**	**PNI covariate definition**	**Sample size (% women)**	**Age (year) (mean or median)**	**Follow-up (months)**	**Events for analysis**
Kawata et al. (14)	Japan	Retrospective cohort study	AHF	Continuous	141 (46.8%)	84	12.0	MACEs
Ju et al. (15)	China	Retrospective cohort study	AHF	Continuous	8,893 (55%)	81	3.0	All-cause mortality
Çinier et al. (16)	Turkey	Retrospective cohort study	HFrEF	Quartiles	1,100 (20.5%)	60.9	48.0	All-cause mortality
Sze et al. (17)	UK	Prospective cohort study	CHF	Continuous Malnourished (PNI ≤38.0)	467 (33.0%)	76.0	18.5	All-cause mortality MACEs
Candeloro et al. (18)	Italy	Prospective cohort study	AHF	Continuous Malnourished (PNI ≤34)	344 (54.1%)	84.0	5.3	All-cause mortality
Alataş et al. (23)	Turkey	Retrospective cohort study	AHF	Malnourished (PNI ≤41.2)	628 (53.7%)	74.7	NA	All-cause mortality
Zencirkiran and Kahraman (24)	Turkey	Prospective cohort study	HFpEF	Malnourished (PNI < 37.0)	285 (54.4%)	68.0	12.0	MACEs
Chien et al. (25)	China	Retrospective cohort study	HFpEF	Malnourished (PNI < 38.0)	1,120 (60.6%)	77.2	41.8	All-cause mortality MACEs
Takikawa et al. (26)	Japan	Prospective cohort study	AHF	Malnourished (PNI < 38.0)	457 (46.6%)	79.0	12.0	All-cause mortality
Sze et al. (27)	UK	Prospective cohort study	CHF	Malnourished (PNI < 38.0)	3,386 (39.0%)	75.0	52.4	All-cause mortality
Shirakabe et al. (28)	Japan	Retrospective cohort study	AHF	Malnourished (PNI < 38.0)	458 (34.0%)	76.0	12.0	All-cause mortality
Cheng et al. (29)	China	Prospective cohort study	AHF	Continuous Malnourished (PNI ≤44.8)	1,673 (32.0%)	76.0	31.5	All-cause mortality
Sze et al. (30)	UK	Prospective cohort study	AHF	Malnourished (PNI ≤ 38.0)	265 (38.0%)	82.0	19.9	All-cause mortality
Narumi et al. (20)	Japan	Prospective cohort study	CHF	Malnourished (PNI ≤ 38.0)	388 (40.0%)	69.6	28.4	MACEs

*AHF, acute heart failure; CHF, chronic heart failure; HF, heart failure; HFrEF, heart failure with preserved ejection fraction; HFpEF, heart failure with preserved ejection fraction; MACEs, major adverse cardiac events; NA, not available; PNI, prognostic nutritional index*.

### PNI and Risk of All-Cause Mortality in Patients With HF

**Figure 2:**
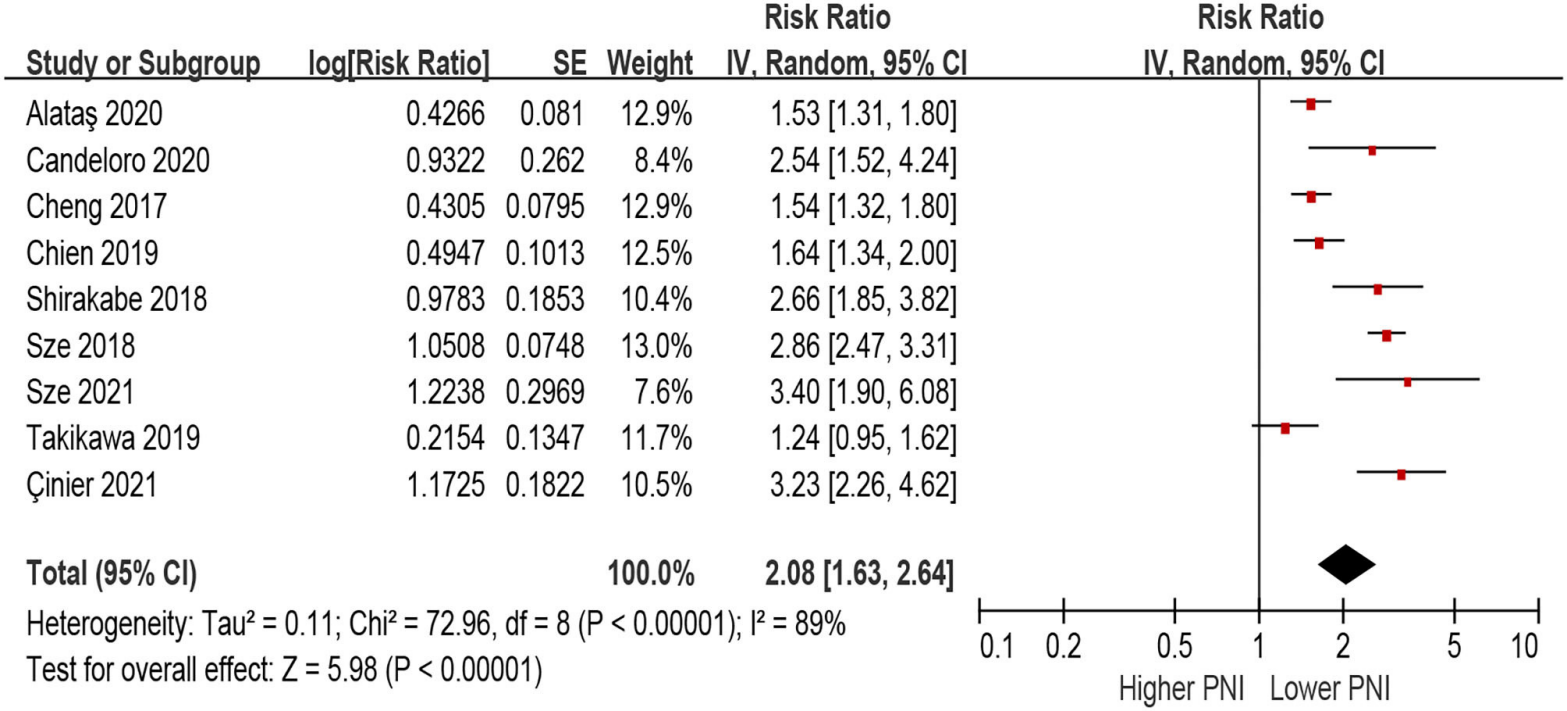
The association between PNI (defined as lower vs. higher) and the risk of all-cause mortality in HF (unadjusted model). CI, confidence interval; HF, heart failure; PNI, prognostic nutritional index.

**Figure 3:**
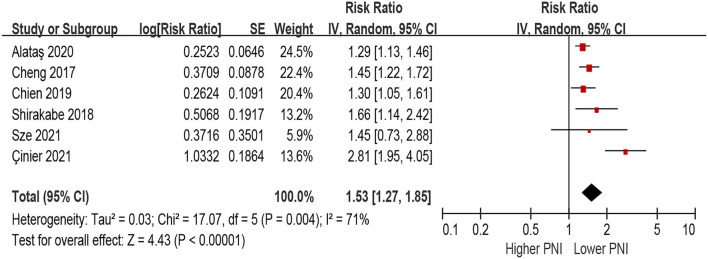
The association between PNI (defined as lower vs. higher) and the risk of all-cause mortality in HF (multivariable-adjusted model). CI, confidence interval; HF, heart failure; PNI, prognostic nutritional index.

In the unadjusted model, patients with lower PNI (malnourished) were associated with a 108% increase in all-cause mortality (HR 2.08, 95% CI 1.63–2.64; *I*^2^ = 89%, *P* for heterogeneity <0.001), compared with those with higher PNI (normal nutritional status; Figure 2). In the multivariable-adjusted model, the association between lower PNI and risk of all-cause mortality was still with statistical significance (HR 1.53, 95% CI 1.27–1.85; *I*^2^ = 71%, *P* for heterogeneity = 0.004; Figure 3).

**Figure 4:**
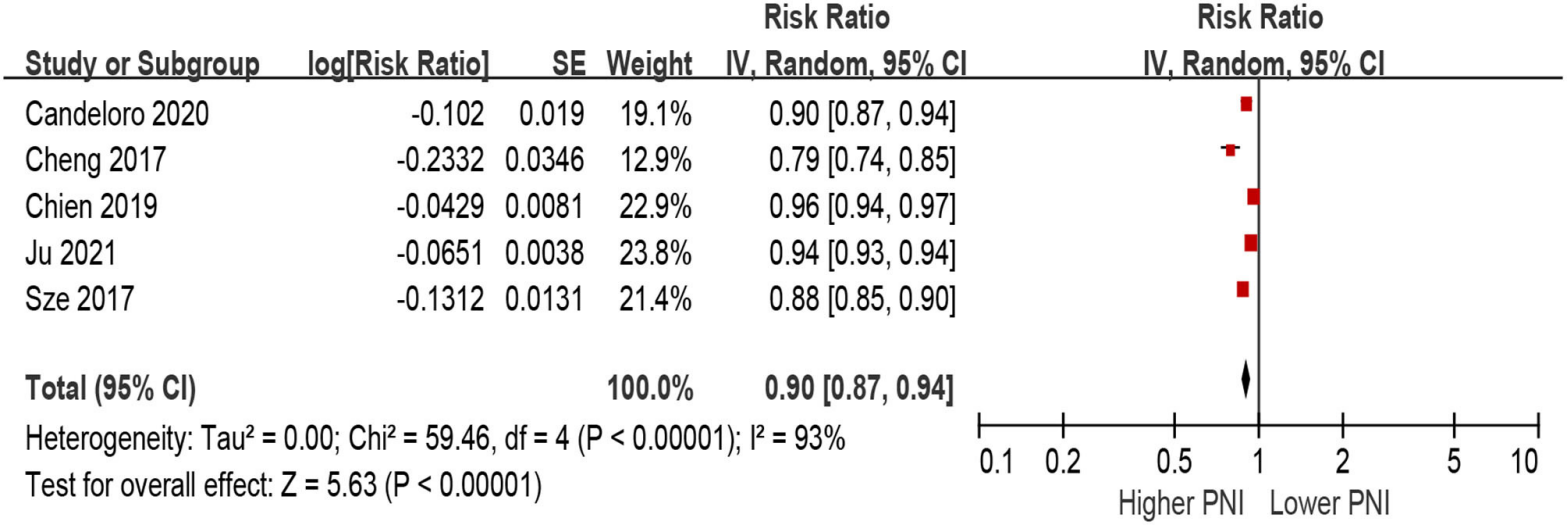
The association between PNI (defined as per 1 increment) and the risk of all-cause mortality in HF (unadjusted model). CI, confidence interval; HF, heart failure; PNI, prognostic nutritional index.

**Figure 5:**
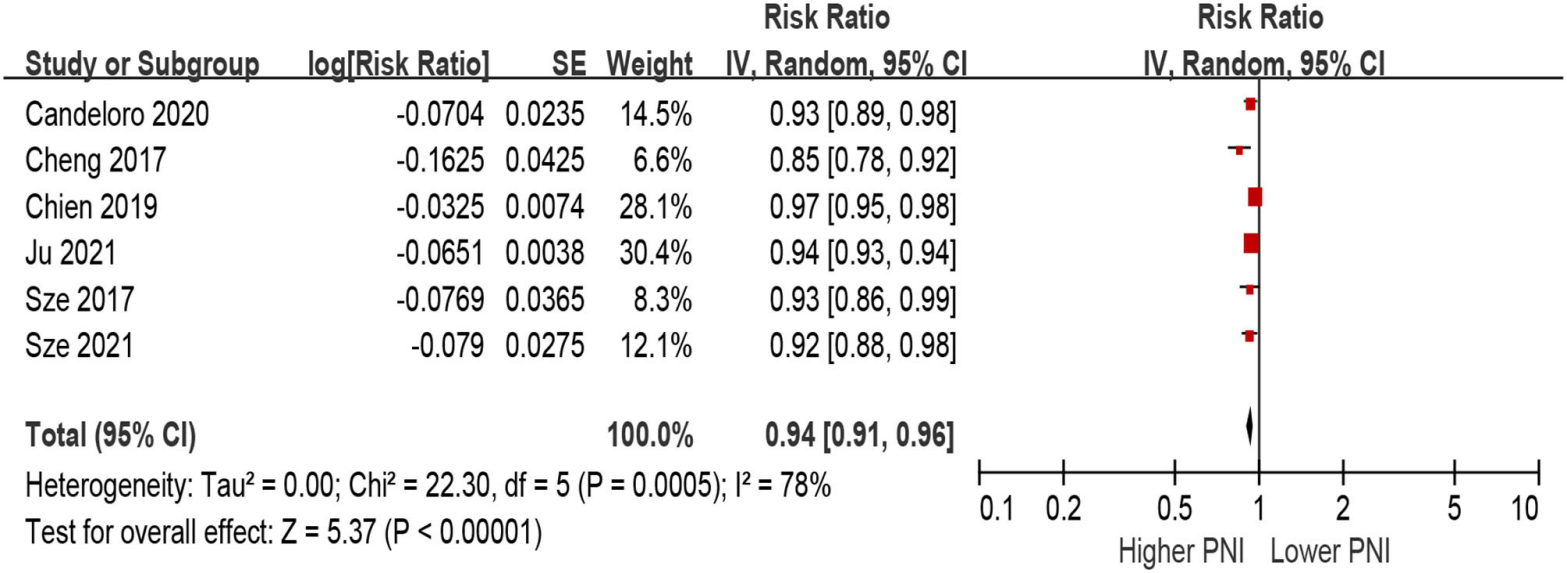
The association between PNI (defined as per 1 increment) and the risk of all-cause mortality in HF (multivariable-adjusted model). CI, confidence interval; HF, heart failure; PNI, prognostic nutritional index.

When PNI was defined as per 1 increment as a continuous metric, we observed that higher PNI was associated with a decrease in all-cause mortality in the unadjusted model (per 1 increment of PNI: HR 0.90, 95% CI 0.87–0.94; *I*^2^ = 93%, *P* for heterogeneity <0.001; Figure 4), as well as in the multivariable-adjusted model (per 1 increment of PNI: HR 0.94, 95% CI 0.88–0.96; *I*^2^ = 78%, *P* for heterogeneity <0.001; Figure 5).

### PNI and Risk of MACEs in Patients With HF

**Figure 6:**
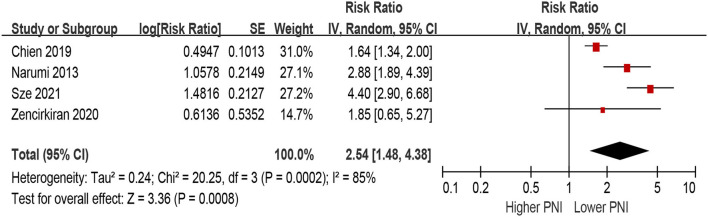
The association between PNI (defined as lower vs. higher) and the risk of major adverse cardiac outcomes (MACEs) in HF (unadjusted model). CI, confidence interval; HF, heart failure; MACEs, major adverse cardiac outcomes; PNI, prognostic nutritional index.

**Figure 7:**
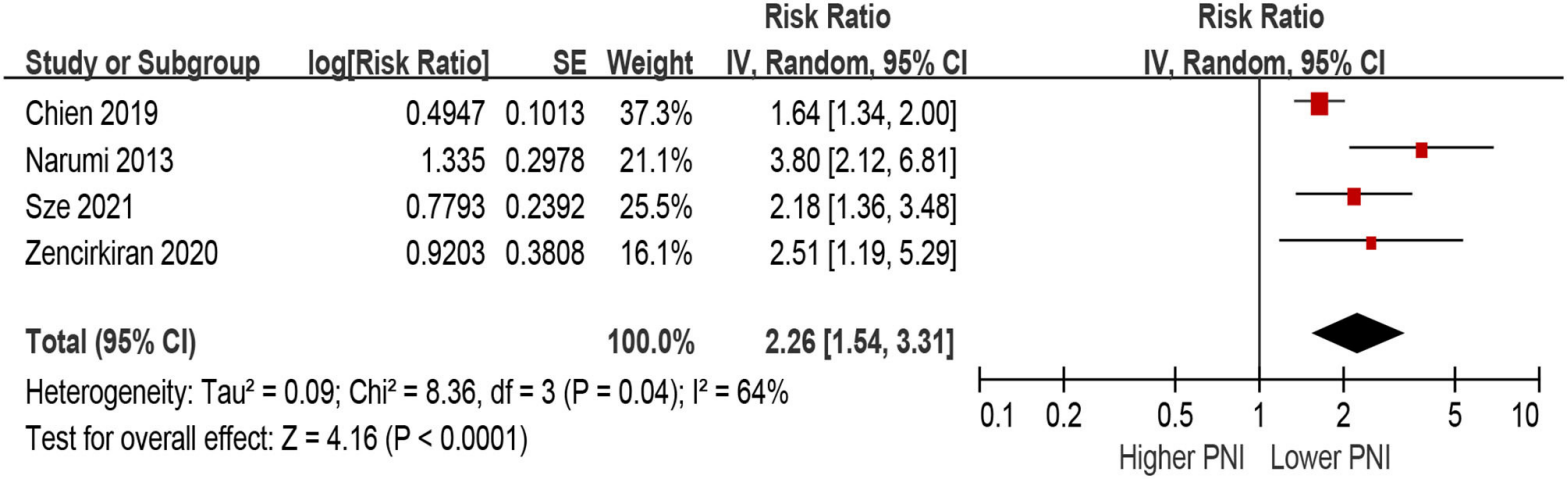
The association between PNI (defined as lower vs. higher) and the risk of MACEs in HF (multivariable-adjusted model). CI, confidence interval; HF, heart failure; MACEs, major adverse cardiac outcomes; PNI, prognostic nutritional index.

Patients with lower PNI were associated with a 154% increase in MACEs (HR 2.54, 95% CI 1.48–4.38; *I*^2^ = 85%, *P* for heterogeneity <0.001), compared with those with higher PNI in the unadjusted model (Figure 6). In the multivariable-adjusted model, lower PNI was still associated with an increased risk of MACEs (HR 2.26, 95% CI 1.54–3.31; *I*^2^ = 64%, *P* for heterogeneity = 0.04; Figure 7).

Only two studies defined PNI as per 1 increment as a continuous metric and evaluated the association between the risk of MACEs. Higher PNI was associated with a decrease of MACEs in the unadjusted model (per 1 increment of PNI: HR 0.95, 95% CI 0.91–1, Supplementary File 3), as well as in the multivariable-adjusted model (per 1 increment of PNI: HR 0.97, 95% CI 0.95–0.98, Supplementary File 4).

### Sensitivity Analyses and Publication Bias Evaluation

Our sensitivity analyses confirmed that the association of PNI with the risks of all-cause mortality and MACEs did not change with the use of fixed-effects models for the random-effects models or recalculation of the relative risks by omitting one study at a time in the meta-analysis. We did not observe significant publication bias for all-cause mortality or MACEs associated with PNI by visual inspection of the funnel plot (Supplementary Files 5–12).

## Discussion

To the best of our knowledge, this is the first meta-analysis evaluating the prognostic effect of PNI in patients with HF. We found that PNI can serve as an important biomarker in predicting a worse prognosis, including all-cause mortality and/or re-hospitalization in HF, either used as a continuous level or a category metric for defining the nutritional status. Although attenuated, the association between PNI and prognosis of HF was still significant after adjustment for multiple confounders, indicating that the prognostic effect of PNI was independent of other established risk factors.

Malnutrition is common in patients with HF and has been proposed as a modifiable risk factor for improving the prognosis in these patients (31). Nutritional intervention in HF can improve the life quality and reduce the risk of mortality (32). Therefore, it is important to evaluate the nutritional status and aid in risk stratification and treatment strategies in the clinical setting. Some multi-dimensional nutritional screening tools, including the Nutritional Risk Screening (NRS-2002), and Mini Nutritional Assessment–Short Form (MNASF) have been developed for evaluating the nutritional risks in patients with HF (33, 34). However, these tools include multiple clinical indexes and dietary factors extracted by questionnaires, which are time-consuming to perform, with limited generalizability in routinely use, and may be confounded by recall bias from the patients.

In contrast, the PNI is a simple metric that can be easily calculated from serum albumin level and lymphocyte count, which are available in most clinical laboratories. Lower PNI (malnutrition) in patients with HF may be contributed to both reduced albumin and lymphocyte count. The components of the PNI, serum albumin, and lymphocyte count, may indicate independent prognostic information in patients with HF. Reduced serum albumin levels may be influenced by malnutritional status or an indication of renal and liver dysfunction. Low lymphocyte count could indicate immune hypo-responsiveness and increased cortisol levels, which could harm the prognosis of HF. Therefore, a lower PNI is not only a metric of malnutrition but also an indication of pro-inflammation status, which provides objective information on “malnutrition-inflammation” complex syndrome for determining increased mortality in HF (33, 34). Similar to PNI, some other simple blood-based biomarkers have also been used to assess the nutritional status in HF, such as the controlling nutritional status (CONUT) score (25, 35), and geriatric nutritional risk index (GNRI) (36, 37). However, the CONUT score is calculated from serum albumin, lymphocyte count, and cholesterol, which may be confounded by the broad use of statin in patients with HF. The GNRI requires body weight for calculation, which will be confounded by fluid overload status and dramatic change in HF treatment during hospitalization. Therefore, the PNI may be a more suitable index that can be easily calculated using routine laboratory parameters and offers a practical tool for evaluating the nutritional risk of HF. However, which index is better for predicting the prognosis in HF needed further exploration. Furthermore, the change of PNI during hospitalization would be important to evaluate the association between this metric and the prognosis of HF. Kawata et al. (14) showed that the changes in PNI on admission and at discharge during hospitalization, were associated with 1-year mortality in patients with acute HF. These findings further support the use of PNI to guide the risk stratification in HF.

Some limitations should be noted in the current study. First, the type and definition of HF were different in the included studies, which may contribute to the clinical heterogeneity. Second, the underlying inflammation status was not adjusted in most of the included studies. Therefore, it is difficult to attribute the prognostic effect of PNI to only malnutritional status or a combination of inflammation status. Third, we did not have individual patient data to perform the comprehensive subgroup analysis. Fourth, the cut-point for malnutritional status with PNI was different in the included studies, which would be an important heterogeneity among the studies.

In conclusion, PNI can serve as an easily calculated bedside “malnutrition-inflammation” biomarker in HF. Lower PNI was associated with a worse prognosis in patients with HF. Future studies are needed to explore whether treatment targeting improving the PNI can provide a positive effect on the prognosis of HF.

## Data Availability Statement

The original contributions presented in the study are included in the article/Supplementary Material, further inquiries can be directed to the corresponding author.

## Author Contributions

J-DW, J-QL, M-YC, and J-XW: research idea and study design. M-YC, J-XW, M-TL, and X-YJ: data acquisition. J-DW, X-LW, Z-WX, and J-QL: data analysis/interpretation. X-LW and Z-WX: statistical analysis. J-DW and J-QL: supervision and mentorship. All authors contributed important intellectual content during manuscript drafting or revision and accept accountability for the overall work by ensuring that questions pertaining to the accuracy or integrity of any portion of the work are appropriately investigated and resolved.

## Funding

J-DW was supported by the Guangdong Basic and Applied Basic Research Fund (Key project of Guangdong-Foshan Joint Fund) (2019B1515120044). The funders had no role in the study design, data collection, data analysis, data interpretation, or writing of the report.

## Conflict of Interest

The authors declare that the research was conducted in the absence of any commercial or financial relationships that could be construed as a potential conflict of interest.

## Publisher's Note

All claims expressed in this article are solely those of the authors and do not necessarily represent those of their affiliated organizations, or those of the publisher, the editors and the reviewers. Any product that may be evaluated in this article, or claim that may be made by its manufacturer, is not guaranteed or endorsed by the publisher.

## References

[1] Ziaeian B Fonarow GC. Epidemiology and aetiology of heart failure. Nat Rev Cardiol. (2016) 13:368–78. 10.1038/nrcardio.2016.25 26935038 PMC4868779

[2] Cai X Liu X Sun L He Y Zheng S Zhang Y . Prediabetes and the risk of heart failure: a meta-analysis. Diabetes Obes Metab. (2021) 23:1746–53. 10.1111/dom.14388 33769672

[3] Heidenreich PA Bozkurt B Aguilar D Allen LA Byun JJ Colvin MM . 2022 AHA/ACC/HFSA guideline for the management of heart failure: executive summary: a report of the American College of Cardiology/American Heart Association Joint Committee on Clinical Practice Guidelines. J Am Coll Cardiol. (2022) 79:1757–80. 10.1016/j.jacc.2021.12.011 35379504

[4] Benjamin EJ Muntner P Alonso A Bittencourt MS Callaway CW Carson AP . Heart disease and stroke statistics-2019 update: a report from the American Heart Association. Circulation. (2019) 139:e56–28. 10.1161/CIR.0000000000000659 30700139

[5] Mai L Wen W Qiu M Liu X Sun L Zheng H . Association between prediabetes and adverse outcomes in heart failure. Diabetes Obes Metab. (2021) 23:2476–83. 10.1111/dom.14490 34227220

[6] Li W Huang A Zhu H Liu X Huang X Huang Y . Gut microbiota-derived trimethylamine N-oxide is associated with poor prognosis in patients with heart failure. Med J Aust. (2020) 213:374–9. 10.5694/mja2.50781 32959366

[7] Wu J Qiu M Sun L Wen J Liang DL Zheng S . alpha-Linolenic acid and risk of heart failure: a meta-analysis. Front Cardiovasc Med. (2021) 8:788452. 10.3389/fcvm.2021.788452 35059448 PMC8764440

[8] Yang S Chen H Tan K Cai F Du Y Lv W . Secreted frizzled-related protein 2 and extracellular volume fraction in patients with heart failure. Oxid Med Cell Longev. (2020) 2020:2563508. 10.1155/2020/2563508 32454934 PMC7229555

[9] Wu J Zheng H Liu X Chen P Zhang Y Luo J . Prognostic value of secreted frizzled-related protein 5 in heart failure patients with and without type 2 diabetes mellitus. Circ Heart Fail. (2020) 13:e7054. 10.1161/CIRCHEARTFAILURE.120.007054 32842761

[10] Hu Y Yang H Zhou Y Liu X Zou C Ji S . Prediction of all-cause mortality with malnutrition assessed by nutritional screening and assessment tools in patients with heart failure: a systematic review. Nutr Metab Cardiovasc Dis. (2022). 10.1016/j.numecd.2022.03.009 35346547

[11] McDonagh TA Metra M Adamo M Gardner RS Baumbach A Böhm M . 2021 ESC guidelines for the diagnosis and treatment of acute and chronic heart failure. Eur Heart J. (2021) 42:3599–726. 34447992 10.1093/eurheartj/ehab368

[12] Kubota K Ito R Narita N Tanaka Y Furudate K Akiyama N . Utility of prognostic nutritional index and systemic immune-inflammation index in oral cancer treatment. BMC Cancer. (2022) 22:368. 10.1186/s12885-022-09439-x 35392843 PMC8991673

[13] Murnane LC Forsyth AK Koukounaras J Pilgrim CHC Shaw K Brown WA . Low muscularity increases the risk for post-operative pneumonia and delays recovery from complications after oesophago-gastric cancer resection. Anz J Surg. (2021) 91:2683–9. 10.1111/ans.17203 34580983

[14] Kawata T Ikeda A Masuda H Komatsu S. Changes in prognostic nutritional index during hospitalization and outcomes in patients with acute heart failure. Heart Vessels. (2022) 37:61–8. 10.1007/s00380-021-01888-x 34131778

[15] Ju C Zhou J Lee S Tan MS Liu T Bazoukis G . Derivation of an electronic frailty index for predicting short-term mortality in heart failure: a machine learning approach. ESC Heart Fail. (2021) 8:2837–45. 10.1002/ehf2.13358 34080784 PMC8318426

[16] Çinier G Hayiroglu MI Pay L Yumurtaş AÇ Tezen O Eren S . Prognostic nutritional index as the predictor of long-term mortality among HFrEF patients with ICD. Pacing Clin Electrophysiol. (2021) 44:490–6. 10.1111/pace.14170 33438766

[17] Sze S Pellicori P Zhang J Weston J Clark AL. The impact of malnutrition on short-term morbidity and mortality in ambulatory patients with heart failure. Am J Clin Nutr. (2021) 113:695–705. 10.1093/ajcn/nqaa311 33236050

[18] Candeloro M Di Nisio M Balducci M Genova S Valeriani E Pierdomenico SD . Prognostic nutritional index in elderly patients hospitalized for acute heart failure. ESC Heart Fail. (2020) 7:2479–84. 10.1002/ehf2.12812 32588975 PMC7524259

[19] Stroup DF Berlin JA Morton SC Olkin I Williamson GD Rennie D . Meta-analysis of observational studies in epidemiology: a proposal for reporting. Meta-analysis Of Observational Studies in Epidemiology (MOOSE) group. JAMA. (2000) 283:2008–12. 10.1001/jama.283.15.2008 10789670

[20] Narumi T Arimoto T Funayama A Kadowaki S Otaki Y Nishiyama S . Prognostic importance of objective nutritional indexes in patients with chronic heart failure. J Cardiol. (2013) 62:307–13. 10.1016/j.jjcc.2013.05.007 23806549

[21] Wells GA Shea B O'Connell D Peterson J Welch V Losos M . The Newcastle-Ottawa Scale (NOS) for assessing the quality of nonrandomised studies in meta-analyses. [serial online]. Available online at: http://www.ohri.ca/programs/clinical_epidemiology/oxford.asp (accessed January 1, 2008).

[22] Zheng S Qiu M Wu J Pan XF Liu X Sun L . Long-chain omega-3 polyunsaturated fatty acids and the risk of heart failure. Ther Adv Chronic Dis. (2022) 13:374130192. 10.1177/20406223221081616 35321400 PMC8935400

[23] Alataş ÖD Biteker M Yildirim B Acar E Gökçek K. Comparison of objective nutritional indexes for the prediction of in-hospital mortality among elderly patients with acute heart failure. Eur J Emerg Med. (2020) 27:362–7. 10.1097/MEJ.0000000000000690 32217850

[24] Zencirkiran AH Kahraman S. Prognostic nutritional index predicts one-year outcome in heart failure with preserved ejection fraction. Acta Cardiol. (2020) 75:450–5. 10.1080/00015385.2019.1661139 31498720

[25] Chien SC Lo CI Lin CF Sung KT Tsai JP Huang WH . Malnutrition in acute heart failure with preserved ejection fraction: clinical correlates and prognostic implications. ESC Heart Fail. (2019) 6:953–64. 10.1002/ehf2.12501 31400092 PMC6816066

[26] Takikawa T Sumi T Takahara K Kawamura Y Ohguchi S Oguri M . Prognostic importance of multiple nutrition screening indexes for 1-year mortality in hospitalized acute decompensated heart failure patients. Circ Rep. (2019) 1:87–93. 10.1253/circrep.CR-18-0018 33693118 PMC7890280

[27] Sze S Pellicori P Kazmi S Rigby A Cleland JGF Wong K . Prevalence and prognostic significance of malnutrition using 3 scoring systems among outpatients with heart failure: a comparison with body mass index. JACC Heart Fail. (2018) 6:476–86. 10.1016/j.jchf.2018.02.018 29753673

[28] Shirakabe A Hata N Kobayashi N Okazaki H Matsushita M Shibata Y . The prognostic impact of malnutrition in patients with severely decompensated acute heart failure, as assessed using the Prognostic Nutritional Index (PNI) and Controlling Nutritional Status (CONUT) score. Heart Vessels. (2018) 33:134–44. 10.1007/s00380-017-1034-z 28803356

[29] Cheng YL Sung SH Cheng HM Hsu PF Guo CY Yu WC . Prognostic Nutritional Index and the risk of mortality in patients with acute heart failure. J Am Heart Assoc. (2017) 6:e004876. 10.1161/JAHA.116.004876 28649089 PMC5669149

[30] Sze S Zhang J Pellicori P Morgan D Hoye A Clark AL. Prognostic value of simple frailty and malnutrition screening tools in patients with acute heart failure due to left ventricular systolic dysfunction. Clin Res Cardiol. (2017) 106:533–41. 10.1007/s00392-017-1082-5 28204965

[31] Qian Y Qian X Shen M Vu A Seres DS. Effect of malnutrition on outcomes in patients with heart failure: a large retrospective propensity score-matched cohort study. Nutr Clin Pract. (2022) 37:130–6. 10.1002/ncp.10815 34994478

[32] Wickman BE Enkhmaa B Ridberg R Romero E Cadeiras M Meyers F . Dietary management of heart failure: DASH diet and precision nutrition perspectives. Nutrients. (2021) 13:4424. 10.3390/nu13124424 34959976 PMC8708696

[33] Joaquin C Alonso N Lupon J de Antonio M Domingo M Moliner P . Mini Nutritional Assessment Short Form is a morbi-mortality predictor in outpatients with heart failure and mid-range left ventricular ejection fraction. Clin Nutr. (2020) 39:3395–401. 10.1016/j.clnu.2020.02.031 32169324

[34] Tevik K Thurmer H Husby MI de Soysa AK Helvik AS. Nutritional risk screening in hospitalized patients with heart failure. Clin Nutr. (2015) 34:257–64. 10.1016/j.clnu.2014.03.014 24755236

[35] Kato T Yaku H Morimoto T Inuzuka Y Tamaki Y Yamamoto E . Association with Controlling Nutritional Status (CONUT) Score and in-hospital mortality and infection in acute heart failure. Sci Rep. (2020) 10:3320. 10.1038/s41598-020-60404-9 32094392 PMC7039945

[36] Yasumura K Abe H Iida Y Kato T Nakamura M Toriyama C . Prognostic impact of nutritional status and physical capacity in elderly patients with acute decompensated heart failure. ESC Heart Fail. (2020) 7:1801–8. 10.1002/ehf2.12743 32410337 PMC7373881

[37] Matsumura K Teranaka W Taniichi M Otagaki M Takahashi H Fujii K . Differential effect of malnutrition between patients hospitalized with new-onset heart failure and worsening of chronic heart failure. ESC Heart Fail. (2021) 8:1819–26. 10.1002/ehf2.13279 33655718 PMC8120416

