# Supplementary material for: Association Between Prognostic Nutritional Index and Prognosis in Patients With Heart Failure: A Meta-Analysis
Source: Front Cardiovasc Med. 2022 Jun 10;9:918566. doi: 10.3389/fcvm.2022.918566 (PMC9226429; doi:10.3389/fcvm.2022.918566)
Supplement: Supplementary file 1 [file Data_Sheet_1.doc]

**Supplementary file 1**. **Confounders adjusted in the included studies**

| **Study** | **Confounder adjusted** |
| --- | --- |
| Kawata 2022 | NA |
| Ju 2021 | Age, neutrophil-to-lymphocyte ratio, male sex, depression, parkinson’s disease, arthritis, paranoia, skin ulcer, pneumonia, falls, skin and soft tissue infection, mycoses, gout, urinary tract infection, charlson score |
| Çinier 2021 | Age, gender, comorbidities (diabetes, heart failure, chronic obstructive pulmonary disease, cerebrovascular accident, and atrial fibrillation), indication for the implantation, device types, laboratory parameters (hemoglobin, creatinine, urea, ALT, and glucose), and the LVEF. |
| Sze 2021 | Age, BMI, AF, NYHA (III/IV compared withI/II), Charlson score, log[NT-proBNP], Hb, and eGFR. |
| Candeloro 2020 | Age, sex, bedridden, anaemia, and NT-proBNP. |
| Alataş 2020 | Age, sex, Log NT-proBNP, coronary artery disease |
| Zencirkiran 2020 | Age, NYHA class, NT-proBNP，albumin, C-reactive protein |
| Chien 2019 | Age, BMI, sex, prior heart failure, hypertension, cardiovascular disease, diabetes, SBP, heart rate, and AF, hyperlipidaemia, eGFR, and BNP |
| Takikawa 2019 | NA |
| Sze 2018 | NA |
| Shirakabe 2018 | Age, systolic blood pressure, heart rate, creatinine, total bilirubin, sodium, C-reactive protein, hemoglobin, left ventricle ejection fraction |
| Cheng 2017 | Age, sex, eGFR, left ventricular ejection fraction, serum sodium level, systolic blood pressure, use of b-blocker, renin–angiotensin system blockade, and spironolactone, NT-proBNP |
| Sze 2017 | NA |
| Narumi 2013 | Age, gender, NYHA class, and plasma BNP levels |

AF, atrial fibrillation; BMI: body mass index; BNP: brain natriuretic peptide; eGFR: estimated glomerular filtration rate; HF: heart failure; LVEF: Left ventricular ejection fraction; NYHA: New York Heart Association; NT-proBNP, N-terminal pro-B-type natriuretic peptide; SBP: systolic blood pressure

**Supplementary file 2. Quality Assessment of the Included Studies**

| **Study** | **Selection**  **(stars awarded)** | **Comparability (stars awarded)** | **Outcome**  **(stars awarded)** | **Quality (total stars)^*^** |
| --- | --- | --- | --- | --- |
| Kawata 2022 | 2 | 1 | 2 | Fair (5) |
| Ju 2021 | 3 | 2 | 3 | Good (8) |
| Çinier 2021 | 3 | 2 | 2 | Good (8) |
| Sze 2021 | 2 | 1 | 2 | Good (7) |
| Candeloro 2020 | 4 | 1 | 2 | Fair (6) |
| Alataş 2020 | 3 | 2 | 3 | Good (8) |
| Zencirkiran 2020 | 3 | 2 | 3 | Good (8) |
| Chien 2019 | 2 | 1 | 2 | Good (7) |
| Takikawa 2019 | 3 | 1 | 2 | Fair (6) |
| Sze 2018 | 3 | 1 | 2 | Fair (6) |
| Shirakabe 2018 | 3 | 2 | 3 | Good (8) |
| Cheng 2017 | 2 | 1 | 2 | Good (7) |
| Sze 2017 | 2 | 1 | 2 | Fair (6) |
| Narumi 2013 | 2 | 1 | 2 | Good (7) |

**^*^** Included studies were graded in quality as good if awarded with ≥7 stars or fair if 4-6 stars.


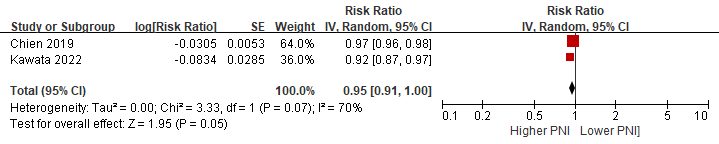


**Supplementary file 3. The association between PNI (defined as per 1 increment) and the risk of MACE in HF (unadjusted model)**

CI, confidence interval; HF, heart failure; MACEs, major adverse cardiac outcomes


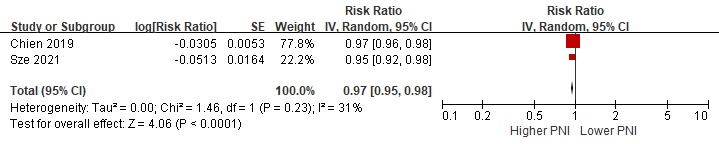


**Supplementary file 4. The association between PNI (defined as per 1 increment) and the risk of MACE in HF (multivariable adjusted model)**

CI, confidence interval; HF, heart failure; MACEs, major adverse cardiac outcomes


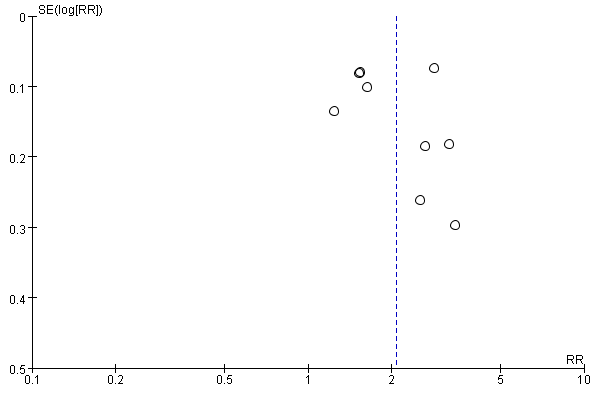


**Supplementary file 5. Funnel plot of comparison: The association between PNI (lower PNI vs higher PNI) and the risk of all-cause mortality in HF (unadjusted model)**

**
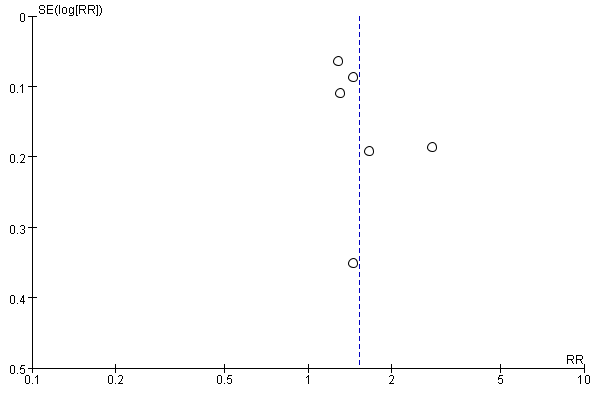
**

**Supplementary file 6. Funnel plot of comparison: The association between PNI (lower PNI vs higher PNI) and the risk of all-cause mortality in HF (multivariable adjusted model)**

**
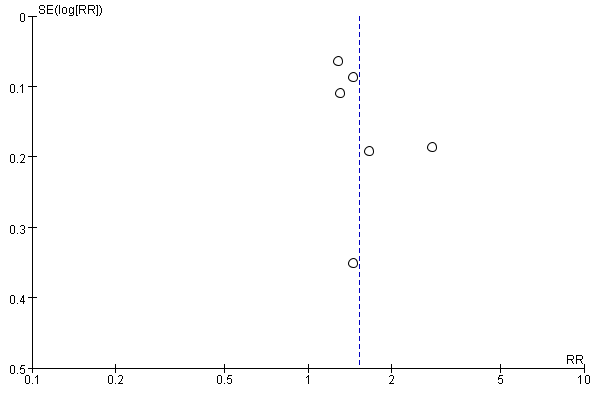
**

**Supplementary file 7. Funnel plot of comparison: The association between PNI (per 1 increment) and the risk of all-cause mortality in HF (unadjusted model)**


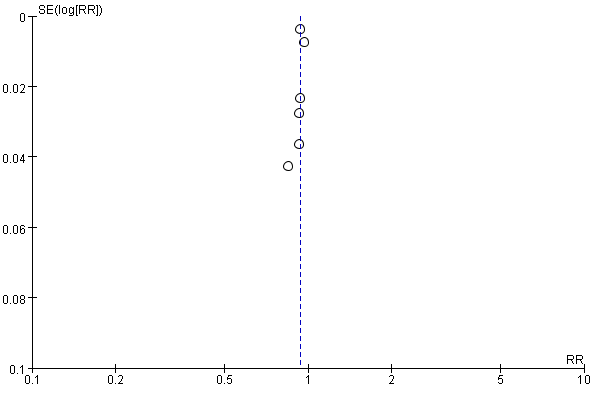


**Supplementary file 8. Funnel plot of comparison: The association between PNI (per 1 increment) and the risk of all-cause mortality in HF (multivariable adjusted model)**


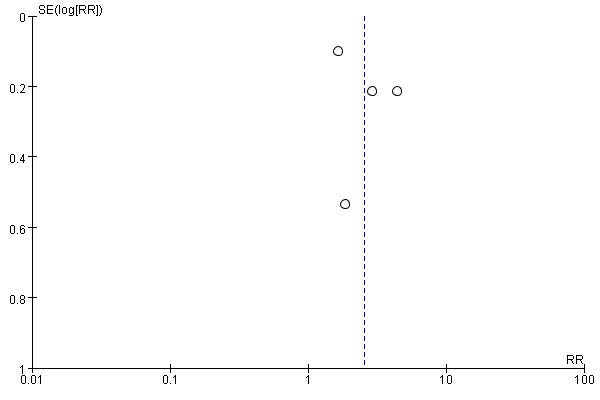


**Supplementary file 9. Funnel plot of comparison: The association between PNI (lower PNI vs higher PNI) and the risk of MACE in HF (unadjusted model)**


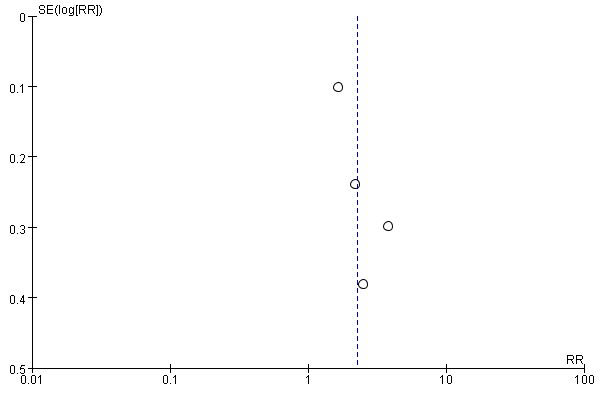


**Supplementary file 10. Funnel plot of comparison: The association between PNI (lower PNI vs higher PNI) and the risk of MACE in HF (multivariable adjusted model)**


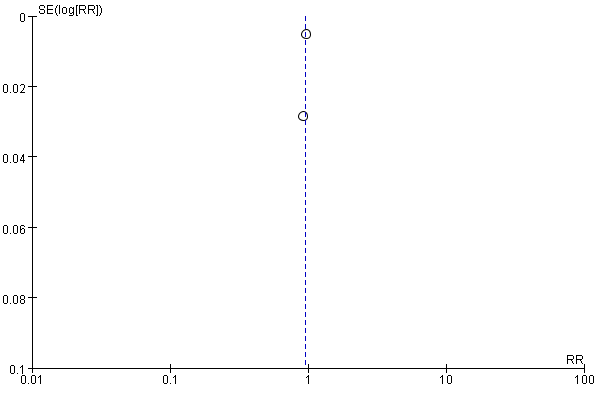


**Supplementary file 11. Funnel plot of comparison: The association between PNI (per 1 increment) and the risk of MACE in HF (unadjusted model)**


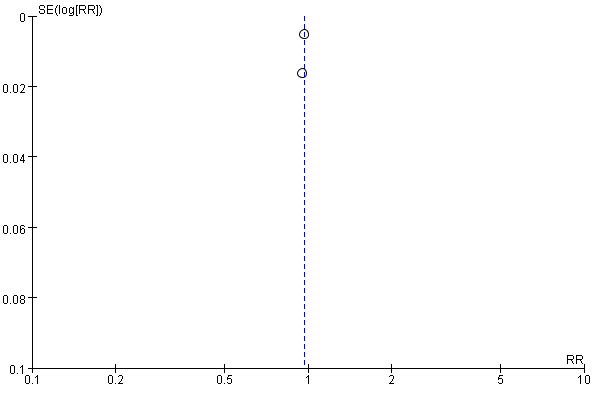


**Supplementary file 12. Funnel plot of comparison: The association between PNI (per 1 increment) and the risk of MACE in HF (multivariable adjusted model)`**
